# Ethical and Scientific Unity in the Medical Profession

**Published:** 1871-03

**Authors:** E. M. Moore

**Affiliations:** Rochester, N. Y.


BUFFALO

### Medical and Surgical Journal.

VOL. X. MARCH, 1871. No. 8.

### Original Communications.

ART. I.—*Ethical and Scientific Unity in the Medical Profession. An Address before the Graduates of the Medical Department of the University of Buffalo, of the Class of* 1871. By Prof. E. M. MOORE, M. D., of Rochester, N. Y.

Another year brings us before our Class for a few parting words. The regiment is the same; the recruits are new. This thought leads me to reflect upon the construction of that army to which we belong, and which has held its march down the ages, varying its plans under the light of new acquisitions of knowledge, but still the same army, animated with like purposes and controlled with a similar *esprit du corps*. This is true, whether the leadership be that of Hipprocrates, struggling with the plague of Athens, during the Peloponnesian war; or of the school of Salernum, which gave its care to the Crusaders retiring from the heats and pestilence of the East; or, that of our own day, when the profession offers its services to every one, even the pauper and the criminal—and is to be found in the van-guard, with the missionary, seeking the improvement of savage life.

What is the bond that holds us together? "The struggles for existence, and the survival of the fittest," plays their part constantly and well. There is no escape from their operation. Man daily and hourly deals with the problem. The bears and bulls of the prairie and forest, tear each other with no more remorse than their compeers of the city. This law is universal, controlling daily life, from the contest of boys who vend you the daily paper, to the Franco-German war; for I suppose it must be conceded that, through all the thin disguises of Hohen-Zollern princes, and future succession to the Spanish throne, the real cause of the apparently causeless war, was one of this most profound, and I may say most justifiable of all causes for war, namely, to settle the question,—who is the better man,—a German or a Frenchman.

But all around this fierce display of forces is seen something which modifies and softens these extreme results: the thought that the less fit may survive, blossoms out in charity, and the law of concord demands such rules as may repress the violence incidental to this fundamental law, and hence organization to soften its asperity. Therefore, in former times, the men who pursued trades were set off in guilds with rules. At present our divisions are more natural.

To what branch then of inquirers can you be said to belong? Obviously to those who pursue the study of natural science. Their methods must be your methods; their failings must be your failings; their triumphs must be your triumphs. Whatever may be said in future times of the poverty in the mechanical and scientific processes of to-day, this century must ever be regarded as the era of natural science. In these less than a hundred years, the world as seen by the dullest vision—has made strides in those arts which contribute to the power of man—in a ratio that is geometrical, as compared with any antecedent period. This is so trite, that I should not have so repeated it, but for the sake of illustration. What then are the special processess that science demands? The careful collection of facts, their association by some common bond of agreement, and the verification of their relations. This, as a matter, of course, abnegates authority; and the scientific inquirer must look constantly to the verification of the facts. The breadth of inquiry is commensurate with the universe: no subject is too remote or too sacred to be reached. In its broadest sense, and in the literal one, science means knowledge; but it is a knowledge of the *kosmos*. Hence its pursuit must be endless. But inquiry seeks to unravel its mysteries, and place itself in relation with the divine at any point most accessible to reach. Hence, one picking a stone from a quarry, asks himself numerous questions. Whence came this here? How was it formed? What is the difference of its various constituents? And, what are the elements which make up the ultimate forms? What is, also, its relation to the stone on the neighboring hill? and why are they so unlike the stones which lie above and below them?

These infantile questions have been asked, undoubtedly, from the beginning of time; but is is not until they have been partially answered, that scientifie inquiry has begun. It is not until numerous facts, susceptible of association, have been accumulated, that this inquiry can be said to have been fairly established. These accumulations are enormous, before any definite relations can be shown as existing amongst them. When these relations are shown; when the crystal, under certain relations, always assumes a definite form; and when, higher still, the ultimate particles are shown to unite in definite proportions, then a science is born—then we have arrived at the divine; then we think as God thinks—the last reach of human inquiry; we see with the eyes of God; we have propounded his law, and the thick vail of darkness, at this point, is entirely rent. Well may it be said, that a new science is born; for, here, our knowledge of the kosmos is perfected.

Thus, at any point of inquiry, where the facts have had their association, perfected into law, we give them a special name. Hence, you have the sciences of geology, of chemistry, of anatomy, of mineralogy, of physiology, and so on through the long list. Perhaps the one most remarkable in the enormous accumulations of its details, with the least apparent results, is meteorology. Through a whole century have patient men daily recorded the condition of the weather. "The wind bloweth where it listeth, and thou hearest the sound thereof, but canst not tell whence it cometh or whither it goeth." But only to-day have these been combined to show how the great storm sweeps the continent; and practical man, by his telegraphic triumph, for the benefit of the mariner and husbandman, placards on the mart whence the wind cometh and whither it goeth.

In the earliest history of medicine, which has come down through Greek channels, we discover its condition in this country, which was undoubtedly as far advanced as any other on the globe. We learn that the physician sustained a semi-priestly character, administering his medicine and his regimen under the guidance of a special divinity. These were chiefly bestowed in houses in the neighborhood of temples, or as often as possible at some healthy spot on the shores of the charming Ægean, whose bracing breezes, then as now, restored the languid frame.

In fact, these establishments were a species of water-cure, whose modes differed as much as the orthodox piety of Clifton from the free and easy ways of Dansville.

In the earlier conditions of the world, and in the less favored nations of to-day, the great mass of facts are referred to supernatural agency; and it has only been by slow degrees, and great effort, that the government of law has removed them from a specially divine or demoniacal possession. And this great advance and elevation of the human intellect is chiefly due to natural science.

One religion has displaced another, and yet they all, in their earlier stages, have taught that specially divine interference was the rule of nature. The Greek and the Roman consulted his auger for his omens, and made his sacrifice to the special deity, who could reverse the laws of nature, and for which sacrifice he hoped it might be done.

During that splendid period of Greek history, when the human mind seemed to culminate, when the talent and freedom of thought of the known world blazed forth upon the theatre of Athens, the Father of Medicine arose. Brought up by the Æsclepiadæ, his pure and truthful mind rejecting the incantations, expanded into the scientific method; and that army to which we belong was then organized, and has since held its march down the ages.

This was truly the golden age of Athens. Pericles, in the full development of intellectual life, already past the middle age; Socrates, in the bloom of early manhood; and Hippocrates, nine years younger, constitute a trio such as the world has never seen. Pericles had raised Athens to its highest point of glory, and stands confessed as the greatest statesman of antiquity. Socrates is believed, to-day, to have brought moral and religious truth in closer relation to the elevated standard of Christianity, than any philosopher of antiquity. While Hippocrates is the founder of our profession as now practiced, and hence appropriately styled the Father of Medicine. But what a different position the two contemporaries occupy in the general estimation of to-day. Socrates is in a condition of apotheosis, for in him is shown forth a moral grandeur, as is supposed, vastly beyond his country-men, and for which they substituted hemlock for the cross. But the name of Hippocrates is seldom mentioned, but to point some sneer at the medical profession. He was only a doctor. The metaphysical webs which minds, and fine ones, have always been prone to spin, acquired a most exquisite tenuity, reproducing everything, and more, than is to be found in the pages of Stewart and Reed, before even the Greek language was developed. The data were stated, and the conclusions drawn, by the astute Brahmin, at the time when the Sanscrit was a spoken language. Where does the exponent of physical science stand in the lines of Brahminical caste. Of the four great castes there are various subdivisions, and he occupies the last place in the second caste. His science is represented by his position, and his position will secure him against much progress. Athens, though by no means free from superstitious thraldom, was still the freest place in the then known world, and, as a matter of couse, physical inquiries found their most natural companionship within its walls. Socrates and Hippocrates both laid themselves open to the charge of impiety by their asseveration, that the phenomena of nature were amenable to law and scientifically determinable. Let us now, in the light of the present day, compare the intellectual clearness of those two great men. I now quote that profound historian, George Grote, the best authority on Grecian history:

"Socrates," he says, "distributed phenomena into two classes:—one, wherein the antecedent and consequent was invariable and ascertainable by human study, and therefore future results accessible to a well instructed foresight; the other, and those, too, the most comprehensive and important, which the Gods had reserved for themselves, and their own unconditional agency, wherein there was no invariable or ascertainable sequence, and where the results could only be foreknown by some omen, prophecy or other special inspired communication from themselves."

Each of these classes was essentially distinct, and required to be looked at and dealt with in a manner radically incompatible with the other. Socrates held it wrong to apply the scientific interpretation to the latter, or the theological interpretation to the former.

Physics and astronomy, in his opinion, belonged to the divine class of phenomena, in which human research was insane, fruitless and impious.

On the other hand, Hippocrates merged into one of those two classes of phenomena, the divine and scientifically determinable, which Socrates had put asunder. Hippocrates treated all phenomena as at once both divine and scientifically determinable.

In discussing certain peculiar bodily disorders found among the Scythians, he observes, "The Scythians themselves ascribe the cause of this to God, and reverence and bow down to such sufferers, each man fearing that he may suffer the like; and I, myself, think, too, that these affections, as well as all others, are divine: no one among them is either more divine or human than an other, but all are on the same footing, and all divine; nevertheless, each of them has its own physical conditions, and not one occurs without such physical conditions." In the light of to-day, how much farther was the reach of Hippocrates than that of Socrates; and as, for moral grandeur, he laid this injunction on his students, "So conduct yourselves as if you were the patient and he the physician."

But I again ask, what is the bond which now holds us together? We have discarded the condition of con-fraternity, which held in its various forms the members of the medical profession, from the days of the Æsclepidæ down to the present time.

Dogmatism in medicine has been its bane in times past, dividing it into sects, who regarded each other with the acerbity common to such divisions. But, since the days of Louis, a sect founded on a dogma has ceased to exist in the medical profession; and Broussais was the last of those who controlled medical opinion by their hypotheses. If, then, we have rejected a dogmatic bond, what have we to hold us together? It appears to me that the only cause of co-hesion is an ethical one. And if this fails us, we must go upon the mart with the placard and the advertising dodge.

It is in the nature of progress to reach into the future or the unknown by hypotheses. We must make them as the guides to experiment and observation. But they are of no other possible value until verification has set its seal upon them. Then the condition of hipothesis has ceased, being superseded by fact and law. Since Louis' time the statistical table approximates and foreshadows the law.

The practice of medicine, then, is founded upon the vast accumulation of fact and observation made by mankind. And the physician draws from this great storehouse such materials as his knowledge may control. This, as a matter of course, gives a large range, and each individual is therefore left to an absolute and uncontrolled freedom in his management of disease. Indeed, the liberality of the profession may be said to be perfect—it has culminated.

I cannot better illustrate my position than by comparing the attitude of religious sects towards each other. When the Churchman and Unitarian, the Presbyterian and Universalist, can agree upon a common bond of union, and this broad enough also to take the Catholic, then may you understand the freedom and liberality of the medical profession. Each man is a sect unto himself, leaving nothing but an ethical bond.

The only influence that is brought to bear on us, is such as one mind may exert on another. The force of authority is broken—we possess command of every resource, and applied after any theory. It is all ours: from the iron which strengthens the blood to the spider's web that calms the nerves. We own no exclusive theory.

Let us consider, for a moment, what this bond demands. In the first place, it does not require you to do such things as may enure to your own advantage to the detriment of the community. It is intended to bring out all the knowledge and ability you may possess, and for the benefit of your patient, and at the same time consistent with the rights of your brethren.

Let me illnstrate. A brother practitioner has charge of a case—a severe one—which rouses the sympathy of the community, and has bronght the relatives to the verge of despair. On the waves of rumor comes the evidence, to you, of gross mistakes; and for the sake of a strong case, let us suppose the mistake is real, and for once rumor is correct. Can it be your place to rush to the rescue. Not at all. Who has set you to be a judge over your brother, and what assurance can the relatives have that you are right, and that your brother is wrong? Your officiousness leads to a suspicion of your motives; and, by placing your rival in the wrong, you rouse that pride of opinion which will tempt him not to yield to the obvious dictates of truth. The rule of ethics requires your silence until your opinion is asked. When it is asked, the patient has just one claim upon you, and one only. And that is, to give him the benefit of all your knowledge, and that without reserve. He has a complete and perfect right to it; but he has no right to any judgment on the past. The case is before you, and your duty is with reference to what lies before you. "Whatsoever ye would that men should do unto you, do ye even so unto them." There is your law, and at this point medical ethics sternly demands that you act up to it. To err is human, and this is a failing that physicians are no more exempt from than other men. To whom, says the patient, must you communicate your knowledge?–" I pay you, says he, and demand the statement." But you are not employed to malign your brother. Your duty is perfectly simple: to do what can be done, according to your best judgment, for the existing exigency, and for no other purpose. Only in this way can the best interest of the patient be subserved. In the chaos of emotion that surrounds the sick-bed there is not often that calmness which is necessary to do that which is right; and the strict observance of our rules will bring the greatest safety. Pride of opinion is one of the most subtle of our dangers; and, in consultation, this must be guarded against by the most careful manner. Therefore, such consultations must be absolutely private; and then the freest inter communication obtained. This should never be varied from. By this alone can the best judgment be arrived at.

But, hanging on the skirts of a great army, we find, invariably, the bummer and guerrilla. This was complained of by Hippocrates, and has remained to be the case ever since.

"Quacks have always existed, always will exist, and always ought to exist," once said the distinguished John C. Warren, of Boston, in a medical convention. Half amused, and half surprised at such a statement, in such a place, some of the gentlemen sitting near him asked an explanation of the desirability of such thieves and plunderers. "To take the incurable cases," was his reply. Most physicians of long practice will attest to the relief that is afforded them when treating a case of severe cancer, or advanced phithis, to have the sufferer cease begging him for a straw to save him from going down, but which is to be readily offered by the quack. One of our functions is the practice of what the Greeks styled Euthanasia. Life is limited to us all; and when the organic disease is surely pressing us into the grave, one of our most important duties is to smooth the way, alleviate pain, prolong existence; and science, in its progress, developes many modes of doing this. But, "whatsoever you would that men should do unto you, do ye even so unto them;" and here our rules demand that this is to be applied to our intercourse with the patient. No false hopes of recovery, no flattering tales of success, but the truth. If delirium or mental prostration renders him an improper subject of communication, his next friend must understand the case; and if he prefers the straw stretched forth to him by the medical pirate, your duty is fulfilled. But what of our duties in our intercourse with the guerrilla himself, who refuses our discipline? 'Tis the Uhlan and bummer that gather the plunder without conquering the foe. With these our intercourse must be absolutely *nil.* Under no specious guise can we associate with a quack.

I have already explained to you that the well we draw from contains all—we are not debarred from the use of anything; and when a man cuts himself off from us, he does so by violating such ethics as I have illustrated. All, under whatever name, are narrow and exclusive, and go off from us for purposes of gain, and never in the light of science. Paracelsus lived about the time of the discovery of this continent, and was the King of Quackery, the boldest and most impudent of all. He died at the age of forty-six, after having announced the discovery of the elixir of life, which was to render man immortal. He gave forth the law of "Similia similibus curantea," but the same idea is to be found in the spurious Hippocratic writings. But at present there is no such dogma, and to propound it is a step backwards two thousand years.

They are to be classed under a great variety of names, flaunting their standards, and all burdened with the one cry, "the regulars have failed; and we have the panaceas—simple and safe at all events."

The Hydropath, who washes away the physical sins of the people, uses a remedy that, of course every one knows, stands at the basis of life, and in itself harmless beyond controversy. And then, contrary to the natural instincts which guide to its use, the stomach, skin and blood are drenched into a poverty that results in eruptions and carbuncles, which are hailed with delight as the efforts of nature to expel some disease, and especially the drugs which had been previously swallowed. The Eclectic, more specious, selects from every system, and excludes the noxious from all; but it is a singular circumstance that his exclusion happens to be of those things that are unpopular at the time. There is no system of practice in the sense that is usually understood.

The art rests upon observation and experience, regulated by the sciences that lie at the foundation. No absolute sequences have thus far been discovered in therapeutics, unless we except the appliances of surgery. If a joint is dislocated, you may know that it may be restored. But even the use of an alkali for acidity of the stomach must sometimes be reversed, and an acid substituted. How, then, can whole classes of diseases be attacked in obedience to fanciful expressions of law? There is no creed with its absolute formula. This renders the art more delicate, and calling for knowledge and skill. Almost every one can be taught the general rules of drawing; but is there any law by which a great painter can produce his results with the precision of law. There is no such system. Each physician, as he draws from the great store-house, is, of course, eclectic; but in no such sense as is implied in the draught from systems.

But what of the Homeopath, the pet of the fanciful and rich, who has succeeded in forcing an unusual attention to his dogmas? He claims more education and cultivation than the ordinary quack; and, indeed, it must be confessed that the ranks of this form of quackery have been chiefly recruited from the unsuccessful members of the profession. It would be idle to follow him in his tortuous absurdities—a microscope cannot see them; and they have been. held up enough to undeceive any who does not desire deception The last stage in which it has manifested itself, meets the view of a growing belief. With the solemnities of a scientific experiment, the medicine is placed in a hermetically sealed vial and placed in the hand. Observations are now made and noted. They are decided and marked, as compared with the condition during the absence of the medicated attenuation. How, exclaims the observer, can this produce its effect, except by spiritual impression? Nothing is too attenuated for this.

You will be constantly importuned to meet these men in consultation; and for obvious reasons this is much desired by them.

You cannot touch pitch without danger. If any one could fall into the depth of absurdity implied by their statements, and still be a member of the profession, you may pity his follies; and, if he is not committed to an open attack on the profession, and his pride of opinion is not excited to blind him, he may be reformed. But it must be remembered that the whole doctrine of homeopathy is founded on a dogma; and in order to place themselves upon a reputable footing, its followers have invented the term Allopathy, which means that the profession, as we understand it, is conducted upon a dogma exactly the reverse. This, to all who care to investigate, is a patent falsehood. But your rich neighbor is sick. With the assurance that the rich American so often has, that his rapidly acquired fortune has conferred on him knowledge; and with the most sublime ignorance of every thing but money-making, he demands your attendance with the quack, who has been cunning enough to flatter his vanity. "I don't care for Dr. Johnson—I pay my money and that is enough." Now, you have a right; and it is also your duty to avoid the man who parades himself as your enemy, and vaunts his superiority and direct opposition. But, leaving out these considerations, and admitting his integrity, if you are both honest, a consulta tion cannot be of any benefit to the patient, for, according to his own statement of his views, they are diametrically opposed to yours, and there is no common basis to stand on. If he agrees with your ideas, he is merely a trickster with whom you wish to have nothing to do.

It is often asked, what is the remedy for the abuses into which the community so constantly falls? As these abuses are largely due to the fundamental weaknesses of human nature, there can be no absolute remedy; but, like other abuses, there is some prospect of abatement. Astrology found its abode in the palaces of princes, but astronomy has long since banished it, and now it is practiced and consulted by the most profoundly ignorant alone. The remedy is not to be found in literary culture. This sometimes seems but to sharpen the nerves to the most exquisite of absurdities. I find my remedy in the cultivation of natural science; and I cannot but think that more breadth of views, and solidity of intellect, will be obtained in this way than in any other. There has been, and there is now raging a controversy with reference to the position of scientific studies in a liberal education. The ordinary college corriculum is too short to allow much innovation upon the classical studies. Now, I would not have these reduced. I cannot bear the thought that the wells of language should be closed to educated men. But we live in an age that is especially blessed with the results of scientific inquiry; and the methods that such inquiry demands should be taught the young—and they can be. The name of science should not be a bug-bear; and if the classics must suffer, let them. But, I think, that some knowledge of the ways of the chemist, astronomer, and geologist, should be taught in the common schools. The number of general scholars are necessarily few; and they are inexcusable for the small amount of scientific knowledge they possess. I have looked around among my friends in vain for a man of scientific attainments who seeks aid from a quack. But, of mere literateurs, the number is abundant.

There is nothing more pleasing than to be in contact with men of liberal education; and, by this, I do not mean professional experts, but men whose studies place them *en rapport* with others.

This, I conceive, to be the just meaning of a liberal education. Or, to put it in an exact formula, a liberal education is that which places its possessor in relation to the intellects of great thinkers in all forms of knowledge.

Such education but few possess. Science then must have the *pas*; and, if once begun, it would diffuse itself and leaven the whole lump.

But I must close. May you regard character more than reputation: and I cannot but believe that you will pleasantly solve the law—"of the survival of the fittest."

